# Deep-Learning-Based Coronary Artery Calcium Detection from CT Image

**DOI:** 10.3390/s21217059

**Published:** 2021-10-25

**Authors:** Sungjin Lee, Beanbonyka Rim, Sung-Shick Jou, Hyo-Wook Gil, Xibin Jia, Ahyoung Lee, Min Hong

**Affiliations:** 1 Department of Software Convergence, Soonchunhyang University, Asan 31538, Korea; fijianwa@gmail.com (S.L.); rim.beanbonyka@sch.ac.kr (B.R.); 2 Department of Internal Medicine, Soonchunhyang University Cheonan Hospital, Cheonan 31151, Korea; ssmri@schmc.ac.kr (S.-S.J.); hwgil@schmc.ac.kr (H.-W.G.); 3 Faculty of Information Technology, Beijing University of Technology, Beijing 100124, China; jiaxibin@bjut.edu.cn; 4 Department of Computer Science, Kennesaw State University, Marietta, GA 30144, USA; alee146@kennesaw.edu; 5 Department of Computer Software Engineering, Soonchunhyang University, Asan 31538, Korea

**Keywords:** calcium detection, coronary artery calcium score CT, resnet-50, VGG, inception resnet V2, deep learning, image classification

## Abstract

One of the most common methods for diagnosing coronary artery disease is the use of the coronary artery calcium score CT. However, the current diagnostic method using the coronary artery calcium score CT requires a considerable time, because the radiologist must manually check the CT images one-by-one, and check the exact range. In this paper, three CNN models are applied for 1200 normal cardiovascular CT images, and 1200 CT images in which calcium is present in the cardiovascular system. We conduct the experimental test by classifying the CT image data into the original coronary artery calcium score CT images containing the entire rib cage, the cardiac segmented images that cut out only the heart region, and cardiac cropped images that are created by using the cardiac images that are segmented into nine sub-parts and enlarged. As a result of the experimental test to determine the presence of calcium in a given CT image using Inception Resnet v2, VGG, and Resnet 50 models, the highest accuracy of 98.52% was obtained when cardiac cropped image data was applied using the Resnet 50 model. Therefore, in this paper, it is expected that through further research, both the simple presence of calcium and the automation of the calcium analysis score for each coronary artery calcium score CT will become possible.

## 1. Introduction

Among the various causes of death, stroke and coronary artery disease (CAD) are the leading global causes of death. Statistics from the World Health Organization (WHO) show that in 2019, approximately 17.9 million people died from cardiovascular disease (CVD), accounting for 32% of global deaths, of which 85% were due to heart attacks and strokes. About three-quarters of these occurred in low- and middle-income countries, and 38% of the 17 million premature deaths (under 70 years of age) from noncommunicable diseases in 2019 were due to cardiovascular disease [1].

CAD is caused by narrowing of the coronary arteries. Since the coronary arteries are blood vessels that supply oxygen, blood, and nutrients to the heart, when the coronary arteries are narrowed, it can cause chest pain (angina), difficulty in breathing, and pain. Thus, it can cause pain, dizziness, nausea, and even heart attack [2]. CAD is usually associated with the individual’s lifestyle and habits. Typically, it can appear in individuals who smoke or drink large amounts of alcohol habitually or regularly, and can also appear in people with high cholesterol or high blood pressure and diabetes. Therefore, through proper diagnosis and early evaluation, it is possible to reduce the risk of the progression of fatal CAD by lifestyle modification.

Coronary artery calcification is a phenomenon in which waste or calcium accumulates and hardens in the blood vessel wall of the coronary artery. Coronary artery calcium (CAC) score, a marker of subclinical coronary atherosclerosis, reflects the cumulative exposure to cardiovascular risk factors over the lifetime, and can inform shared decision-making regarding the use of preventive therapies [3,4]. It can be diagnosed by methods such as treadmill test, radionuclide scan, computed tomography (CT) scan, magnetic resonance imaging (MRI) scan, and coronary angiography [5,6,7]. CAC-CT can be used to score CAC. However, these methods generally require a specialist to manually check all data one-by-one. In addition, in the case of medical images, a radiologist must directly check and input each image using medical software, prior to diagnosis.

**Figure A1:**
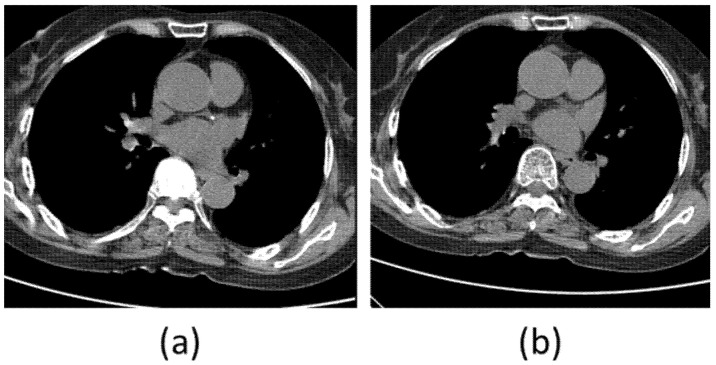
(**a**) CT image with calcification in the artery; (**b**) CT image without calcification.

In this paper, deep-learning is applied to the coronary artery calcium score CT image data to determine the calcification score, which is most commonly used to determine the degree of coronary artery calcification [8]. Appendix A provides a detailed description of coronary artery calcium score CT images and Figure A1 show about example of CT image. Deep-learning in coronary artery calcium score CT images has played a key role in dividing the organs into meaningful areas by considering the characteristics and information of specific organs among various organs, and performs a supporting function of making medical decisions based on the characteristics of the organs [9,10,11]. In particular, CNN models have been steadily applied for a long time in the field of computer vision [12]. CNN models have now been applied in both computer vision and in various applications, such as natural language processing, hyperspectral image processing, and medical image analysis [13]. A CNN model receives 2D data, and extracts high-level functions and features through many hidden layers. Therefore, to provide physiological signals in CNN models, some research teams are using 2D data that is converted from 1D signals [14].

In this paper, we applied three traditional CNN models (Inception Resnet v2, VGG, and Resnet 50) to train the coronary artery calcium score CT images, to determine whether calcification occurred in the coronary arteries or not. We constructed the training data using PNG image format by division into three types. The first type was the original coronary artery calcium score CT images. The second type was the cardiac segmented images in which only the heart region was cut by applying the recent segmentation technique. The last type was the cardiac cropped images created by dividing the segmented cardiac images into nine, and enlarging them. For these data, the three CNN models mentioned above were applied to determine the presence of calcium. This research was conducted with IRB approval (SCHCA202002025-HE001) for an AI-based cardiovascular disease diagnosis study from Soonchunhyang University Cheonan Hospital to apply the CT images of patients to deep-learning training. For the CT image training data used for deep-learning, about 2400 images of 177 individuals provided by Soonchunhyang University Cheonan Hospital were used. To apply the CNN model to the entire provided data, training was performed by classifying it into 1200 CT images with calcium region, and 1200 CT images containing normal cardiovascular features.

## 2. Related Works

CNN deep-learning model, which has been widely used for image classification, is developing rapidly under the influence of the recent rapid increase in big data and the improvement of the processing speed of GPU hardware, and various deep-learning approaches are being conducted through datasets that have collected various public data and assets [15,16,17]. Chougrad et al. [18] developed a CNN model of the CAD framework to discriminate breast cancer. Deep-learning typically requires massive datasets to train networks of a certain depth. In the case of transfer learning, overfitting can easily occur, but it is an effective method for processing a relatively small dataset. These research efforts show that when training a CNN model, the best results can be obtained with optimal fine-tuning, even in the same public dataset classification.

Xie et al. [19] proposed a lung nodule algorithm that adds tissue, shape, and deep model learning information at the decision stage. This algorithm uses a shape descriptor in Fourier—a texture descriptor based on a gray level co-occurrence matrix—to characterize the heterogeneity of the nodule, and automatically learns the feature expression of the nodule in slice units using a deep convolutional neural network (DCNN).

Ming et al. [7] proposed a dense connected residual network (DCRN) for noise reduction in low-dose CT images, which helps to preserve details, and when removing CT noise, makes more efficient data. Research on these medical images is steadily progressing, and research on how to process a dataset and what features to connect and learn is also receiving a lot of attention. In regard to medical imaging data, it is generally not provided as a public dataset that can be used by all researchers; thus, there is the limitation that it is difficult for general researchers to collect such medical datasets.

Van et al. [9] conducted research on each blood vessel, such as the left anterior descending artery (LAD), the left circumflex artery (LCX), and the right coronary artery (RCA). The dataset for this paper included CT from the national lung screening trial (NLST), PET attenuation correction (ACPET) CT, coronary artery calcium scoring CT (CAC–CT), diagnostic CT of the chest, radiation therapy treatment planning (RadTherapy) CT, and CT examinations from the Jackson Heart Study (JHS) consisting of six types, and they conducted CNN learning for each blood vessel, and then learned and evaluated whether calcification had occurred. The baseline is low-dose chest CT examinations, and it learned NLST data and evaluated CT data, such as CAC-CT and ACPET. A small set of examinations of the respective type supplemented to the baseline are data-specific. Learning was performed by combining one other CT data with NLST, and evaluation was performed with the combined data. For the data-specific examinations, training evaluation was performed by combining different CTs with NLST, just as CAC–CT data was included in NLST data to evaluate CAC–CT. Combined data is a combination of examinations of all available types, and it included all other CT data in the NLST data, and was conducted as a method to evaluate the learning of other CT data.

Martin et al. [20] proposed a study on deep-learning-based automatic coronary calcium score algorithm evaluation with data of 511 patients from 2014 to 2018. All data were collected on a dual-source CT scanner (SOMATOM Force, Siemens Healthineers, Erlangen, Germany). Deep-learning-based automated calcium analysis was performed using the prototype proposed by Martin (Automated Scoring Siemens Healthineers), which is based on a neural network with ResNet architecture and a fully connected neural network for spatial coordinate functions. They used Hounsfield units, and trained on 2000 datasets. Accuracy was decided according to CACS. When it was (0, 1–10, 11–100, 101–400, and >400), the accuracy was (94.9%, 73.5%, 95.6%, 98.3%, and 92.3%), respectively. The limitation of this paper is that most of the data consists of data from normal individuals without calcification.

Kotia et al. [21] proposed a concept called “few-shot learning”. Deep learning is being used in many medical fields, but its limitation is that it is highly dependent on the amount of available data. In order to solve this limitation, the desired information is generated by using a small amount of data. Data augmentation plays an important role in few-shot learning; and in order to utilize it, the quality of the data has to be quite high.

## 3. CNN Models

### 3.1. VGG Network

The VGG network was introduced in a paper published by Oxford researchers in 2014 [13], and took second place in the ILSVRC 2014 competition. Unlike previous networks, the core of the VGG network is to deep-stack 3 × 3, a relatively small convolution filter, and it uses a larger number of layers, such as (11, 13, 16, and 19). When three 3 × 3 filters are stacked, one 7 × 7 convolution layer and the receptive field are the same, but the activation function can be used more, so more nonlinearity can be obtained, and at the same time, the number of parameters can be reduced.

**Figure 1:**
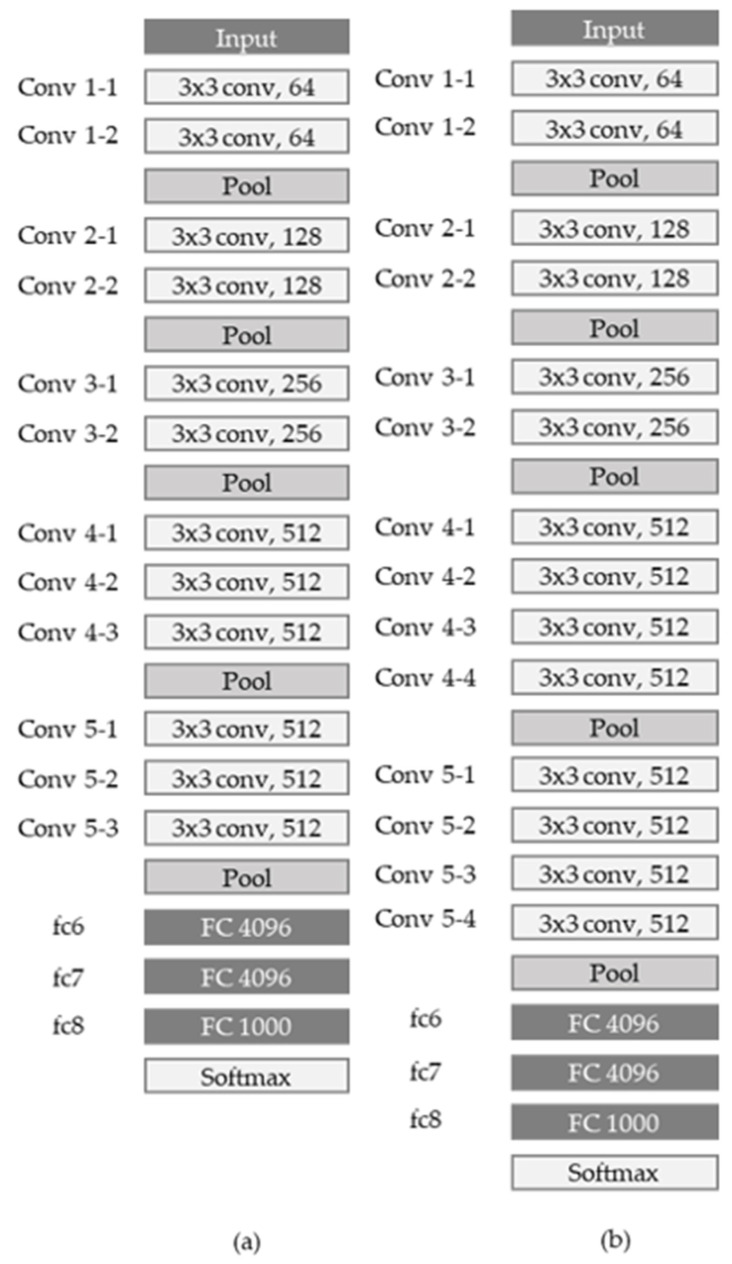
(**a**) Architecture of the VGG16 network; (**b**) Architecture of the VGG19 network.

Figure 1 shows the VGG16 network and VGG 19 network models. Once input data comes in, it is filtered by two convolution layers with 64 3 × 3 kernels and 1 × 1 stride. Then, max pooling with 2 × 2 kernel and 2 × 2 stride is performed, and then it is filtered again into two convolution layers with 128 3 × 3 kernels and 1 × 1 stride. After that, max pooling—which is max pooling through 256 3 × 3 kernels, 512 kernels, and 512 kernels— is performed, and two fully connected layers with 4096 neurons and 1000 fully-connected layers are dense to see if calcification has progressed. It has two output units to determine if it is normal.

### 3.2. Inception Network

**Figure 2:**
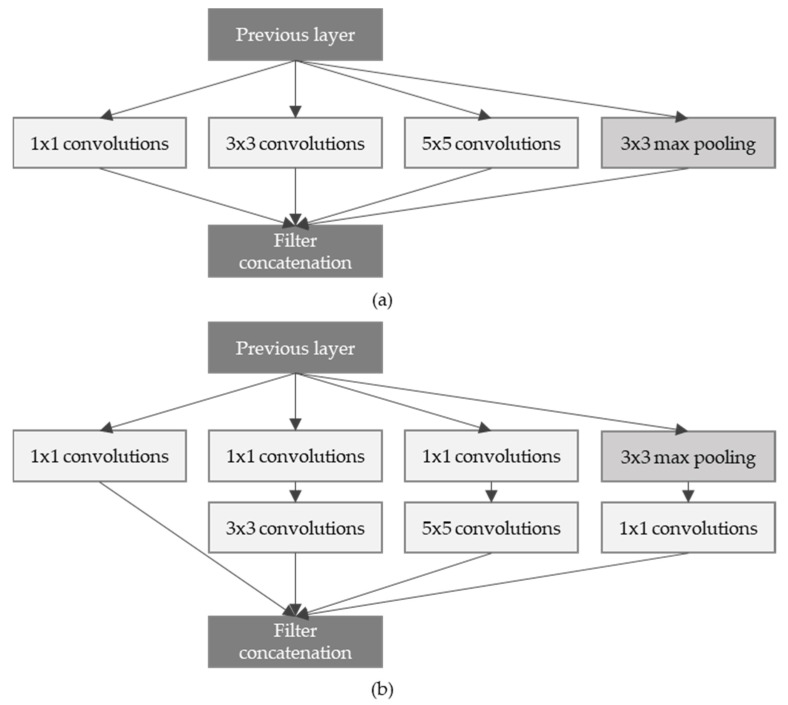
(**a**) Architecture of Inception module in the naïve version, (**b**) Architecture of Inception with dimensionality reduction in the Inception module.

Inception network [22] was announced in the same year as the VGG network, and the model won first place in the ILSVRC 2014 competition. It is composed of a total of 22 layers, and has a very long and complex structure. Inception network proposed a block structure called the Inception module, as shown in Figure 2. The biggest feature is that it uses concatenation, which, after undergoing a total of four different operations, combines the feature maps in the channel direction. In addition, 1 × 1, 3 × 3, and 5 × 5 convolution operations were mixed, and used to express various receptive fields.

This method is called the naïve inception module. In addition, since 3 × 3 convolution and 5 × 5 convolution operations take up a lot of computation, 1 × 1 convolution operation is added before the two convolution operations to reduce the number of feature maps, and then 3 × 3 and 5 × 5 convolution operations are performed to increase the number of feature maps. An Inception module with a dimension reduction method that adds a bottleneck structure that increases was proposed. Due to this, the amount of computation of the inception module can be reduced by more than half.

### 3.3. Residual Neural Network (ResNet)

**Figure 3:**
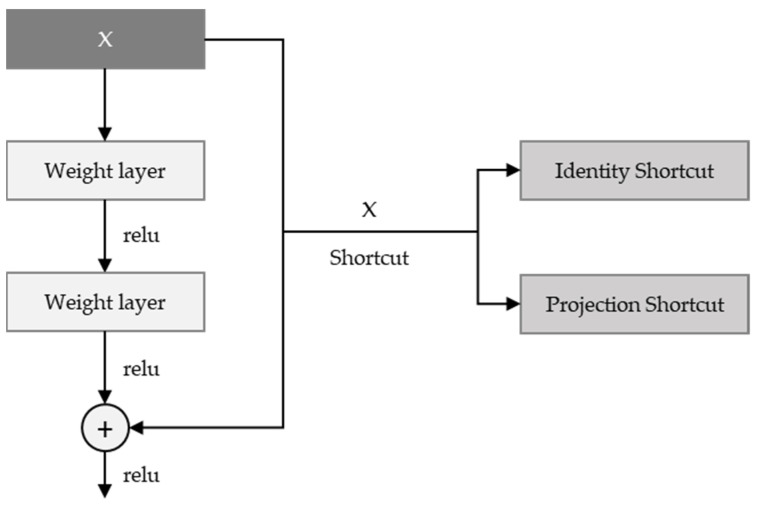
Residual learning: a building block.

ResNet [23] took first place in the ILSVRC 2015 competition with the structure proposed by Microsoft Research. It has a structure similar to VGG, in that the 3 × 3 convolution is repeated. It is classified into ResNet-18, ResNet-34, ResNet-50, ResNet-101, ResNet-152, etc., according to the number of layers. There is a shortcut to the side of the two convolution layers, which is usually an identity shortcut—i.e., adding the input feature map to the output. Whenever the number of output feature maps is doubled, a method of reducing the width and length of the feature map by half is used; and instead of pooling, a convolution operation with stride = 2 is used. In this paper, ResNet-50 is applied, and since the number of feature maps increases in models over 50, the bottleneck structure seen in the inception module is applied to overlap the bottleneck residual block, as shown as Figure 3

## 4. Methodology

### Overall Workflow of the Proposed Method

**Figure 4:**
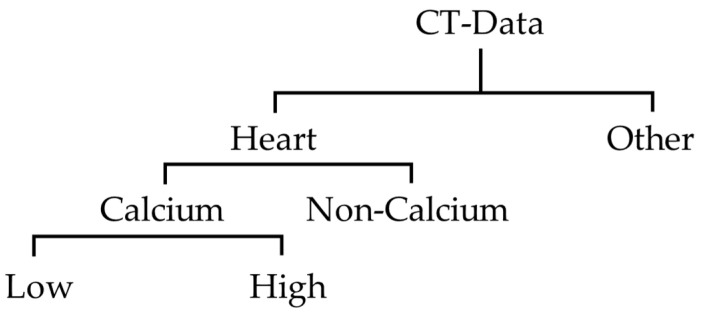
Cardiovascular CT image labeling structure proposed in this paper.

In this research, labeling work was performed on 2400 images of data from 2300 individuals collected at Soonchunhyang University Cheonan hospital. This process was conducted with a radiologist and cardiologist, and anonymization of personal information was applied to all data. The original coronary artery calcium score CT data collected consisted of normal subjects, and those with cardiovascular calcium. For this research, we divided these data into Coronary artery calcium scoring CT with calcium, and without. The coronary artery calcium score CT image data used in this paper were labeled by dividing the heart region and other regions as shown in Figure 4. The images of the heart region were further divided into CT with calcium, and CT without calcium. In addition, when the CCS was lower than 20, the CTs with calcium were classified and labeled as low; and when it was higher than 20, they were labeled as high. This criterion was established because 50% of the collected data had less than 20 points, and 50% had more than 20 points. In the future, and as research progresses, we will use less than 100 as mild plaque burden, and designate less than 10 as minimal plaque burden, and follow the existing classification.

**Figure 5:**
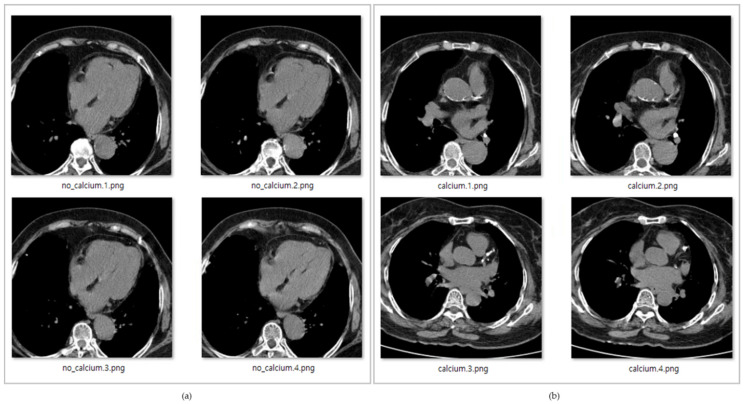
(**a**) Original coronary artery calcium score CT image without calcification, (**b**) Original coronary artery calcium score CT image with calcification.

In Figure 5, the data with title beginning with ‘calcium’ is the data with calcium in the coronary arteries, while the data with title starting with ‘no_calcium’ is the normal data, in which calcification in the coronary arteries has not progressed.

**Figure 6:**
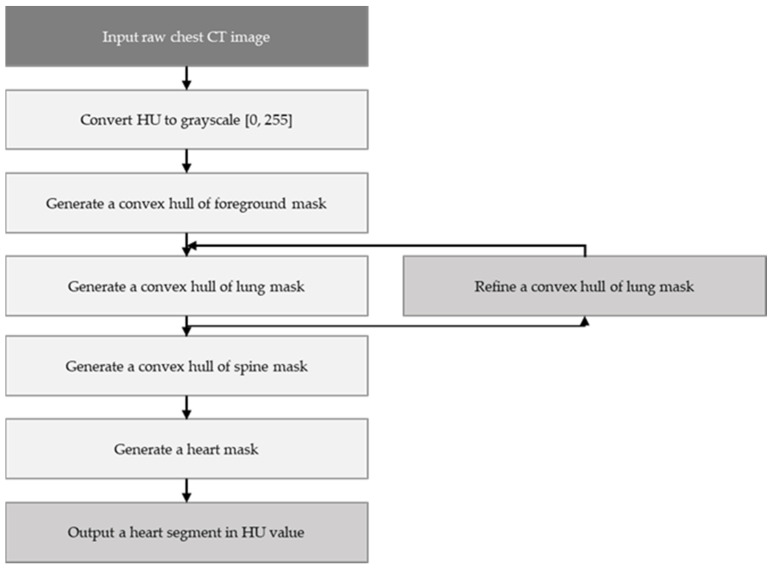
Flowchart for the cardiac region segmentation process.

**Figure 7:**
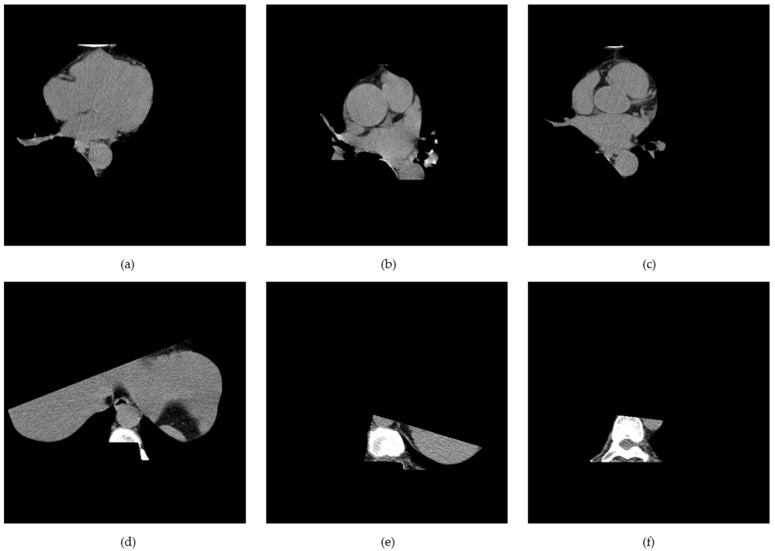
(**a**–**c**) Heart, and (**d**–**f**) non-heart, obtained through the cardiac region segmentation algorithm.

K-means clustering and the mathematical morphology were used to dissect the cardiac region to generate the cardiac segmented images. First, the air material at the edge of the coronary artery calcium score CT image was filtered by the convex hull of the foreground mask. Afterwards, other body organs, such as fat, muscle, and lungs, were processed by the convex hull of the lung mask. Finally, the vertebral part was filtered by the convex hull of the vertebral mask. Figure 6 shows the way in which the whole cardiac segmented images generation process was carried out. The segmentation process is intended to generate a binary mask of the entire cardiac anatomical ROI containing the four chambers, the coronary artery, and the DA. First, the air mass at the edge of the chest CT image is filtered by the convex hull of the foreground mask. Then other body material—such as lungs, muscle, and fat—are filtered by the convex envelope of the lung mask. Finally, the spinal material is filtered by the convex hull of the spinal mask [24]. Figure 7 shows the results of the segmentation process, and in this paper, unnecessary regions in the heart CT image were removed by applying this algorithm.

**Figure 8:**
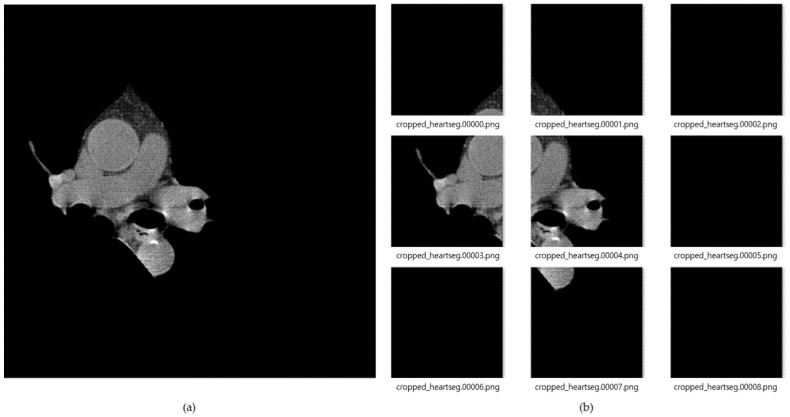
(**a**) Cardiac segmented images, and (**b**) cardiac cropped images created by dividing the segmented cardiac images into 9.

Figure 8 shows the result of images created by dividing the data into nine segments using the cardiac region segmentation data. The image data divided into nine was resized to 299 × 299 pxl size for deep-learning training. General CT images are stored as dcm file format with HU value. In this paper, we applied PNG file format with pixel values. This can be readily used for real-time labeling using a general camera, and as an assistant tool for medical doctors in the future. During data deep-learning training for this image data, there were frequent situations in which training did not proceed, due to lack of memory because of the use of many convolution layers. Therefore, we reduced the batch size to train three CNN models, and applied dropout to prevent overfitting. In the case of training accuracy and training loss, good results were obtained; but in the case of validation, splattered values occurred, due to the lack of training data. To solve this problem, the amount of data was increased by changing the angle, left, right ratio, and position values of the image through the validation generator.

**Figure 9:**
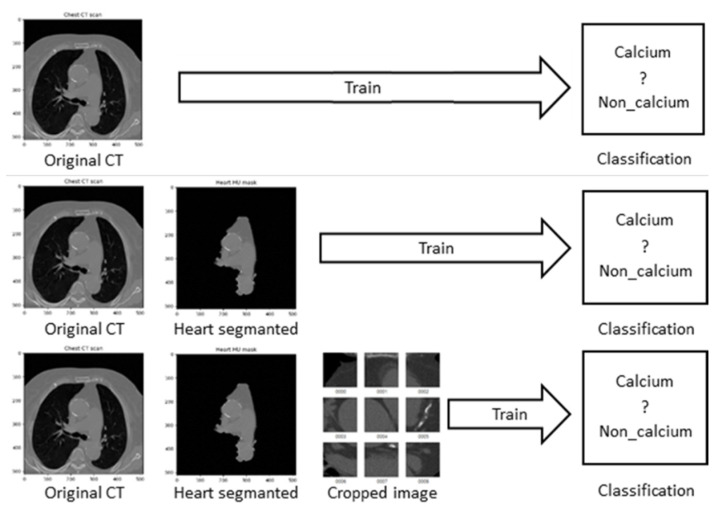
Flowchart of training process for each CT dataset.

To find the optimal model for each image dataset, first, Figure 9 shows that when CT data was input to the current models, the highest performance was evaluated. There are cases in which calcium is formed in many areas of the entire CT image, but otherwise it occupies a very insignificant portion of the total pixels. Therefore, we constructed the most optimal dataset for training and decision by extracting only the necessary areas from the entire learning area.

## 5. Results

### 5.1. Experimental Test Setup

The experimental test in this paper was conducted using Windows 10, Intel Core i7-9700 CPU @ 3.00 GHZ, 32.0 GB RAM, and NVIDIA GeForce RTX 2080 graphics card. The program was written using Python language, and CNN models of Resnet 50, VGG, and Inception Resnet V2, were applied. The optimization was conducted by setting the stochastic gradient descent (SGD) momentum to 0.9, weight decay to 0.005, learning frequency to 0.001, and batch size to 8. Normalization and dropout were performed to prevent overfitting. For each model, our proposed method has 1.46 million parameters for Resnet 50, has 23.53 million parameters for Resnet v2 and has 118.25 million parameters for the VGG, As shown as Table 1. For coronary artery calcium detection from CT images, Resnet 50 was most suitable for our experimental test in number of parameters and inference time.

### 5.2. Data Acquisition

All data used in this paper were collected at Soonchunhyang University Cheonan Hospital, anonymized before use, and were obtained with the approval of the Institutional Review Board (IRB) and Philips iCT 256 with ISP version 10.1 (intelispace portal) were used. The data were used with strict security and applied guidelines. Of the 2300 data collected, 1800 individuals without cardiovascular calcium were normal, while 500 individuals were classified as individuals with cardiovascular calcium. For the collected data, the average CT image data of an individual consists of 56 images, and information on various organs and body materials—such as heart, lungs, ribs, spine, and air—are contained in this image. We used the whole coronary artery calcium score CT data, and at this time, used only the heart region for which it needs to be determined whether or not calcification is in progress based on this, which may be hindered by other organs in learning. Among the collected data, CT data with light bleeding due to a stent or other material was decided to be an outlier, and excluded from the data.

For this paper, the dataset created with these data was divided into the original CT dataset (2400 images), the cardiac segmented CT dataset (1900 images), and the cardiac cropped CT dataset (2800 images). Since general deep learning approach is a data-driven approach, the more data we have, the higher performance we would achieve. Thus, to increase the number of CT data, data augmentation was performed by 10% of each dataset. As a result, the number of original coronary artery calcium score CT images consists of 2200 images for training, 220 for validation, and 220 for testing, for a total 2640 images; the total number of cardiac segmented CT dataset consists of a total 2090 images, with 1760 images for training, 165 images for validation, and 165 images for testing. Finally, the number of cardiac cropped CT dataset consists of a total of 3080 images, with 2420 images for training, 330 images for validation, and 330 images for testing, as shown as Table 2. Due to the cardiac segmentation algorithm, the number of data was reduced, due to the case in which the image was completely erased, and came out as a black image. In the case of the original coronary artery calcium score CT image data, the size was 512 × 512 px, the cardiac segmented CT data was 299 × 299 px, and the cardiac cropped CT data was also processed in the size 299 × 299 px. In this paper, the names of the data files were divided into ‘calcium’ for calcified data and ‘no_calcium’ for non-calcified data, and categorization was carried out through this. Algorithm 1 is a pseudocode that briefly shows the training process for CNN models.
**Algorithm 1:** Pseudocode for training coronary artery calcium score CT images-Train model**Input**: Original coronary artery calcium score CT image dataset, Cardiac segmented image dataset, Cardiac cropped dataset**Output**: Detection of calcification**Begin**1: Read_data (Image_width, Image_height_Image_channel)2: for filename**if** category == “calcium”:Categories.append(1)**else**:Categories.append(0)3: RestNet50 ← load()4: InceptionResNetV2 ← load()5: VGG← load()6: df[‘catefoty’].replace({0: “non_calcium”, 1: “calcium”})7: train_datagen = rescale, shear_range, zoom_range, horizontal_flip, width_shift_range, height_shift_range8: train_generator ← train_datagen.flow_from_dataframe9: validation_generator = validate_datagen.flow_from_dataframe10: model ← create_model()11: **if** training = True **then**12: history13: model.save14: model.evaluate(train_generator, test_generator)15: **return** results()16: **end procedure**

## 6. Discussion

In this paper, we trained the coronary artery calcium score CT image data using Inception Resnet v2, Resnet-50, and VGG models. The procedure was conducted with data from Koreans who visited Soonchunhyang University Cheonan Hospital, and on average, coronary artery calcium score CT images (1 to 56) were collected and applied. When the original coronary artery calcium score CT image data was trained on each model, Resnet-50 showed 88.95% validation accuracy and 81.08% test accuracy; in Resnet v2, the verification accuracy was 52.24%, while the test accuracy was 51.47%; and in VGG, 84.33% and 61.23% values were shown, respectively.

For the cardiac segmented CT image data, in Resnet-50, the validation accuracy was 67.02% and the test accuracy was 81.56%; in Resnet v2, 51.37% validation accuracy and 48.57% test accuracy were obtained; and in VGG, 89.31% and 73.25% values were obtained, respectively. Finally, in Resnet-50, the cardiac cropped CT image data showed 99.08% verification accuracy and 98.52% test accuracy; in Resnet v2, 87.53% verification accuracy and 80.23% test accuracy were obtained; and in VGG, 86.59% and 96.20% values were obtained, respectively. All of the result is in Table 3.

As a result, in the case of the original coronary artery calcium score CT image data and the cardiac segmented CT image data, there was no significant difference in test accuracy in the other models except VGG; while in the validation accuracy, the original coronary artery calcium score CT image data showed higher accuracy in the rest of the models, except VGG. When the cardiac cropped CT image data was used, validation and test accuracy values were higher for all results, except for the validation accuracy of VGG for the cardiac segmented data. Additionally, among CNN models, Resnet-50 showed the highest test accuracy.

In the research of van Velzen et al. [19], baseline showed 95% test accuracy, data-specific provided 97% test accuracy, and combined showed 96% test accuracy. Although their baseline is similar to that of this paper, we did not decide the presence or absence of calcium in each blood vessel, but only the presence or absence of calcium in the whole heart. Therefore, their method can only determine whether calcification has progressed or not, and in this case, the proposed method in this paper shows higher accuracy of 98.52%. In this paper, after deciding the presence or absence of cardiac calcification in the patient, we plan in the future to research each blood vessel with images that have been calcified. This has the advantage of efficiently labeling the presence or absence of calcium when the latest data are collected later.

## 7. Conclusions

In this paper, we divided CT image data into the original coronary artery calcium score CT image dataset, the cardiac segmented image dataset, and the cardiac cropped image dataset, and trained using Resnet-50, Inception Res-net V2, and VGG models. Datasets were stored in PNG format after anonymization through preprocessing. When training the datasets with the three CNN models, when using the cardiac cropped image dataset to determine the presence of calcium, 99.08% verification accuracy and 98.52% test accuracy were obtained, which were higher than those values of the original coronary artery calcium score CT image dataset, or of the cardiac cropped image dataset.

In this paper, only three CNN models were applied to train the coronary artery calcium score CT dataset; however, in the future we plan research to continue the research to find the optimal model and dataset by extending it to all other CNN models. Through this, we plan to find the most appropriate model to determine the presence or absence of calcium in coronary artery calcium score CT image in the future, and conduct research to automate the analysis score for each heart blood vessel, and predict the possibility of disease.

## Figures and Tables

**Table 1:** Computational cost comparison.

Method	No. of Parameters	Inference Time
Resnet 50	1.46 m	24 ms
Resnet v2	23.53 m	74 ms
VGG	118.25 m	102 ms

**Table 2:** Configuration of each dataset.

	Training	Validation	Testing	Total
Original coronary artery calcium score CT images	2200	220	220	2640
Cardiac segmented CT images	1760	165	165	2090
Cardiac cropped CT images	2420	330	330	3080

**Table 3:** Results of validation and test accuracy for each model.

	Validation Accuracy	Test Accuracy
Original coronary artery calcium score CT-data		
Resnet-50	88.95	81.08
Resnet v2	52.24	51.47
VGG	84.33	61.23
Cardiac segmented data		
Resnet-50	67.02	81.56
Resnet v2	51.37	48.57
VGG	89.31	73.25
Cardiac cropped data		
Resnet-50	99.08	98.52
Resnet v2	87.53	80.23
VGG	86.59	96.20

## Data Availability

No new data were created in this study. Data sharing is not applicable to this article.

## Abbreviations

The following abbreviations are used in this manuscript:

CAD	Coronary artery disease
CAC	Coronary artery calcium
CVD	Coronary vascular disease
CNN	Convolutional neural network
CT	Computer tomography
CPU	Central processing unit
LAD	Left anterior descending artery
LCX	Left circumflex artery
RCA	Right coronary artery
MRI	Magnetic resonance imaging
GPU	Graphical processing unit
PNG	Portable network graphics
DCM	Digital imaging and communications in medicine
DCNN	Deep convolutional neural network
WHO	World Health Organization

## Appendix A

Computed tomography (CT) image is a tomography technique in which a subject is placed between an X-ray generator and an X-ray detector, X-ray images called sinograms are taken from various directions, and then restored to a cross-sectional image by calculating inverse radon transformation with a computer. CT is one of the most commonly used medical imaging techniques in the current medical field. Because CT has excellent ability to reproduce cross-sections of the human body, it is possible to determine the degree of deformation of the normal anatomical structure of the human body relatively easily and accurately. It plays a very important role in the field of imaging diagnosis due to the difference in absorption of minute parts by expressing excellent resolution and contrast in distinguishing blood, tissue, cerebrospinal fluid, white matter, gray matter, and tumors. A large amount of calcium pixels appearing in the coronary arteries can be predicted to increase the risk of cardiovascular disease, as shown in Figure A1a:
